# Fine-scale human–flood relationships in the Congo Basin: ethnographic perspectives from the Lac Télé Community Reserve

**DOI:** 10.1098/rstb.2024.0491

**Published:** 2026-08-20

**Authors:** Katherine Meier, Milena Beekmann, Tiriel Lokoka Ngoma, Célie-Léoda Moungouya-Moukassa, Bola Madzoke

**Affiliations:** ^1^ Department of Anthropology, Yale School of the Environment, Yale University, New Haven, CT, USA; ^2^ Department of Biology and Biotechnologies ‘Charles Darwin’, University of Rome La Sapienza, Rome, Italy; ^3^ National School of Agronomy and Forestry, Marien Ngouabi University, Brazzaville, Congo; ^4^ Wildlife Conservation Society, Brazzaville, Congo

**Keywords:** seasonally flooded ecosystems, extreme flooding events, riverine communities, local ecological knowledge, climate change adaptation

## Abstract

The Likouala-aux-Herbes river and surrounding swamp forests of the northern Republic of Congo are described in scientific literature as a wetland forest system characterized by a bimodal flood pulse and widespread seasonal flooding. Recent climatic and hydrological modelling of the Congo Basin and Cuvette Centrale peatlands, moreover, positions the Likouala region as a rain-fed or ombrotrophic peatland. However, human populations experience and adapt to flooding at a more localized scale. This study presents ethnographic data on flooding and seasonality from six villages within the Lac Télé Community Reserve (LTCR), a protected area in the Likouala swamp forest biome and the country's only Community Reserve. Our findings reveal how spatio-temporally variable flooding produces village-specific cultures, livelihood cycles, adaptations and vulnerabilities—an interconnected gradient of biocultural relationships. Across villages, flood dynamics have changed drastically over the last decades. Climate change, economic transitions and erosion of local knowledge systems are straining long-standing adaptive frameworks within and between LTCR communities. We emphasize the importance of ethnographic data in understanding fine-scale human–environment interactions and suggest that biocultural analysis at this scale—what we term the ‘hydrocultural patch’—is essential for effective landscape governance and policy development in the context of accelerating climate change. A French translation of this abstract is available in the supplementary material.

This article is part of the discussion meeting issue ‘African tropical peatlands: function, value and vulnerability’.

## 1. Introduction

The Congo River Basin's hydrology is increasingly recognized for its critical role in the global carbon cycle and the ecosystem services it provides to human populations living in and around it [1,2]. However, the biophysical processes associated with Congo Basin ecosystems remain exceptionally understudied, even as they face escalating pressures from commercial land-use changes (e.g. deforestation and forest degradation from logging, mining and other extractive industries), with the region's wetland forests particularly at risk from climate-change-driven disruptions to seasonal flooding regimes [3]. These combined threats—expanding industrial interests and global climate change—synergize with the lack of baseline environmental data to pose existential risks to the forest-dependent populations (human and non-human) of the region. The spatio-temporal variations in ‘seasonal flooding dynamics, water level variations, and vegetation types’ [2, p. 1857] are among the characteristics of greatest interest to researchers leading the emerging efforts to ‘transform our [scientific] understanding’ of the Congo Basin [2]. Most research prioritizes carbon storage and greenhouse gas dynamics, employing remote sensing and mathematical modelling at a basin-wide scale. While valuable, such approaches often simplify or homogenize ecological complexity and cannot speak to locally relevant biophysical processes occurring at fine scales. These researchers have continuously underscored the need to better account for the ‘heterogeneity of peatland hydrology across the region […] to understand the relationship between the river networks and the adjacent wetlands’, as well as the need to extend ecological valuation of the peatlands to include understanding how they ‘support local livelihoods and cultures’ [3, p. 11].

To address this critical knowledge gap, this study presents fine-scale ethnographic data on local understandings of and adaptations to hydro-climatic variability from six villages of the Lac Télé Community Reserve (LTCR), a protected area in the Likouala Department of the Republic of Congo. By documenting local ecological knowledge, this research aims to elucidate the relational and historical dimensions of hydrological processes, particularly the seasonal flood pulse. Our data, collected through semi-structured interviews and participant observation, provide insights into both village-specific and catchment-wide mechanisms by which communities inhabiting a single river-floodplain wetland observe, interpret and respond to dynamic hydrological processes. Through these data, we highlight the flood-driven diversity in cultures and livelihood cycles of LTCR's human communities, contributing to understandings of peatlands as unique biocultural systems, the conservation of which requires accounting for the entanglements and reciprocity between biophysical and socio-cultural processes [4]. We also emphasize the complexity of local environmental knowledge and value systems across the LTCR, valorization of which is necessary for equitable and sustainable governance solutions [5].

## 2. Methods

### (a) Study site and communities

The Likouala region, part of the Cuvette Centrale depression within the central Congo Basin, is characterized as a seasonally flooded swamp forest. In 2017, the world's largest expanse of tropical peatlands was discovered there [6], catalysing a major uptick in scientific interest and investment in the region. The LTCR is located along a section of the Likouala-aux-Herbes (LAH) river, a tributary of the Oubangui River and part of the broader Congo River system (figure 1).

**Figure 1.**
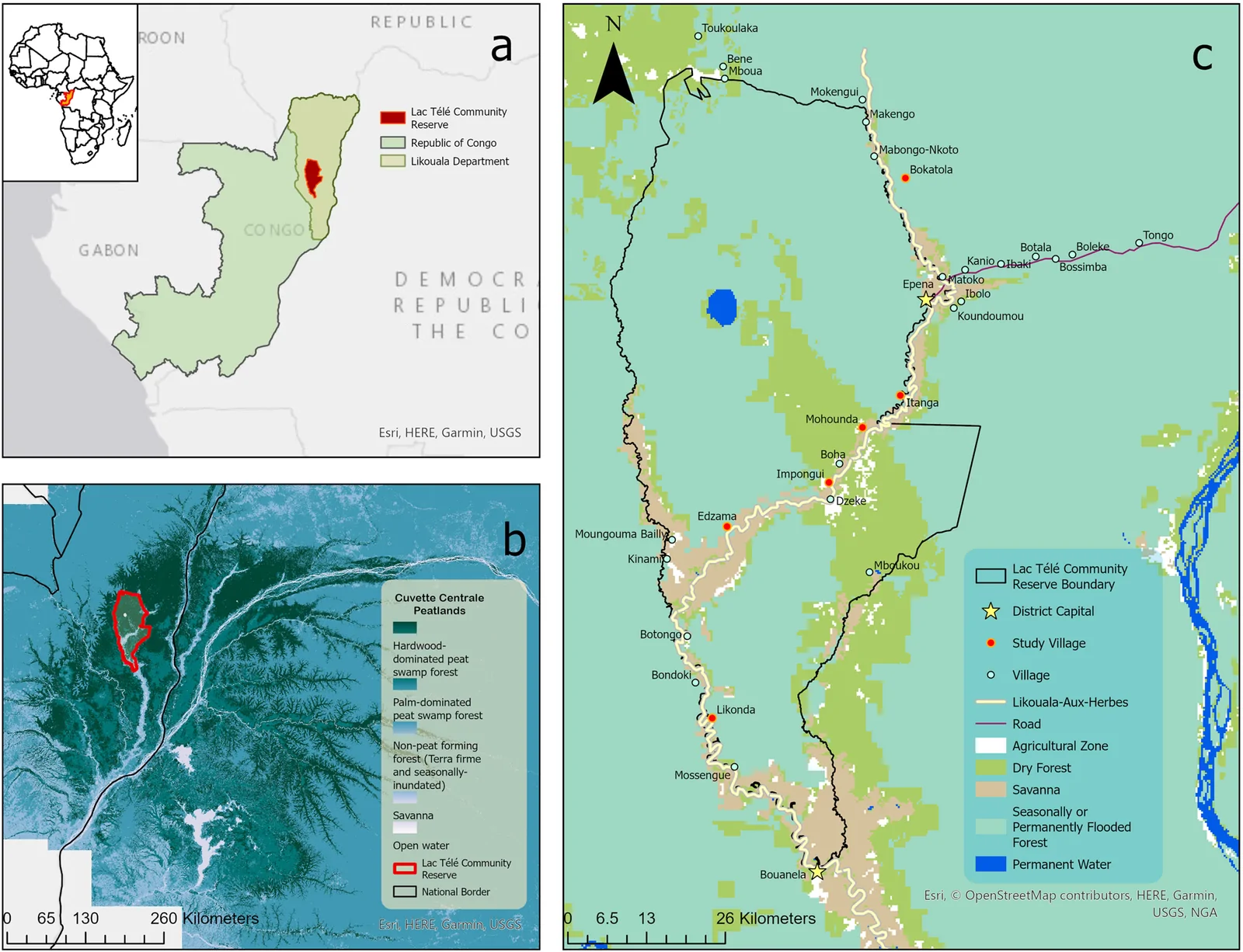
Localization of the study site with ecological zones and village communities. (a) The LTCR located in the Likouala district of the Republic of Congo. (b) Localization of the LTCR within the Congo Basin's Cuvette Centrale peatlands (peatland probability map from Crezee *et al.* [7]. (c) Protected area boundaries, village communities along and around the LAH and broad land-cover classes (ESA GlobCover 2009: https://due.esrin.esa.int/page_globcover.php). Maps produced by K.M

The LAH remains a free-flowing and ecologically intact river-floodplain system, without major anthropogenic modification or development. The river exhibits a bimodal annual flood pulse, creating four distinct seasons commonly referred to as the ‘big’ and ‘small’ rainy and dry seasons. However, past scientific research in the LTCR has often chosen to recognize only ‘low-water’ season and ‘high-water’ seasons (e.g. [8, p. 14]). Annual average rainfall ranges between 1000 and 1800 mm, and the mean annual temperature is 25.6°C [9].

The LTCR was officially gazetted as a protected area in 2001 and is managed by the Congolese Ministry of Forest Economy and the Wildlife Conservation Society (WCS). As the Republic of Congo's only community reserve, the protected area encompasses 27 villages (approx. 20 000 people) distributed from North to South along the riverbank. Throughout most of the Reserve, travel is limited to boat—only one road connects the northern village of Epena to the nearest market centre, Impfondo. Ethnically, the LTCR's population is considered to be functionally homogeneous, with over 90% identified under the umbrella category of Bomitaba, a Bantu group formalized during the French colonial period when small, factious, forest-dwelling clans were relocated to villages along the navigable LAH for easier control and taxation. Much of the remaining 10% of LTCR's population are composed of hunter–gatherer Aka groups as well as immigrants from surrounding regions [8]. The LTCR is ecologically, politically and culturally bisected; ecologically, a northern hardwood swamp-dominated sector and a southern seasonally flooded forest-dominated area are separated by a large, central *terra firma* corridor ([10]; figure 1). Politically, the LTCR spans two administrative districts—Epena and Bouanela—with eponymous village centres, respectively, falling in the northern and southernmost reaches of the LTCR. Though landscape managers qualify the Bomitaba as a unified group, linguistic research and socio-cultural indicators (such as traditional dances and burial rituals) show that those eco-political divides align with cultural subgroup divisions within the Bomitaba, largely between the Reserve's North and South. In the central village of Itanga, for example, residents speak a dialect blending northern and southern variants [11]. Several of our local interlocutors confirmed that individuals from southern villages can have trouble understanding the Bomitaba language spoken in more northern villages and *vice versa*.

LTCR communities depend upon the forest's and river's natural resources for their lives and livelihoods. Living in a wetland ecosystem, LTCR residents have a heavily fish-dependent diet and economy. However, subsistence activities available to residents of each village are diverse and often distinct. Nearly all men engage in a seasonal mix of fishing and hunting. Women also fish and are typically the primary managers of agricultural fields. Fishing rights and access are traditionally governed by customary tenure systems wherein each family maintains exclusive control over designated ponds and specific portions of the river. Agriculture is practised in a traditional swidden cycle, and since the availability of dry land is limited, efforts are heavily concentrated on cassava, the staple starch of the region. Other common crops include plantains, avocados, sugar cane and oil palm. In certain villages, cocoa is also cultivated for commercial purposes. Other subsistence activities, like the gathering of non-timber forest products (NTFPs), are also practised by both men and women: important resources include tree species used in house construction, wild fruits, mushrooms and caterpillars. Several LTCR NTFPs are also important commodities in wider economic trade networks; key among them are smoked fish, cola nuts, honey and dwarf crocodiles. Independent of the permanent Aka communities within the LTCR region, groups of ‘Yaka’ hunter–gatherers migrate seasonally into the LTCR swamp forests from the neighbouring Sangha region where they move between Bomitaba villages in search of these commercially valuable resources (see [12]).

### (b) Data collection

The data used for this study derive from two independent studies conducted between January and December 2023, in six villages: Bokatola, Itanga, Mohounda, Impongui, Edzama and Likonda (figure 1). The two projects were included as part of the doctoral theses of authors K.M., M.B. and T.L.N. Authors K.M. and T.L.N. formed one research team and worked in Impongui, Edzama and Likonda (study one), while authors M.B. and C.-L.M.-M. formed the second and worked in Bokatola, Itanga and Mohounda (study two). Author B.M., a Bomitaba native and site ecologist for WCS, assisted the other authors in the development and realization of their respective field studies. Study one focused on great ape conservation and natural resource use in Reserve local communities, while study two centred around local observations of and adaptation to climate change. As social scientists working contemporaneously on complementary topics, we collaborated to strategically select study villages and avoid overlap while representing the full spectrum of socioecological conditions across the LTCR, including its northern, central and southern zones. Our data collection methods included semi-structured interviews (*n* = 196 informants), focus groups (*n* = 11 groups) and participant observation where we lived in each focal village for six to eight weeks, engaging in the daily life of village communities. After meeting with village chiefs, elders and cultural leaders and gaining approval for work in each village, we employed a snowball sampling approach (study one) or stratified random (study two) to recruit interview respondents who were men and women ages 18 and above. Interviews typically lasted between 1 and 2 h. Focus groups were stratified between men and women and averaged around 20 participants. We asked questions from questionnaires that were pre-approved by the human subject ethics committees of our respective institutions. At the start of each interview, we gained the written or verbal consent of participants after an explanation of the project aims, data management plans, conditions of anonymity, potential participation risks and participation compensation. Interviews were conducted in French, Lingala or Bomitaba, using local interpreters where required. Given the consent of the participant, interviews were recorded on handheld audio recording devices, and we took detailed notes during the interviews.

### (c) Data analysis

Interviews and field notes were transcribed by authors K.M. and T.L.N. for study one and M.B. and C.-L.M.-M. for study two. For each study, transcripts were coded and analysed for major themes. Our respective research objectives collectively encompassed understanding diverse aspects of human–environment relationships, traditional to current-day land use patterns and socioecological changes. We combined data from our two datasets concerning weather, seasonality and flooding and recoded for commonalities and divergences by village. The agentive role of flooding in shaping fine-scale biocultural dynamics across study villages emerged independently in both of our datasets. In the following section, we detail our results and discuss their relevance to ongoing conservation and governance efforts within the LTCR and across the broader peatland forests of the Cuvette Centrale. Village-specific data are distinguished by the village from which they originated.

## 3. Results

### (a) Seasonal flooding dynamics and hydro-climatic variation across the Lac Télé Community Reserve

The regularity of the seasonal flood pulse in river–floodplain systems like that of the LAH is thought to be the driver behind the system's ecological diversity and productivity [13]. Though the LTCR ecosystem at the river catchment level has experienced a regular flood pulse for at least as long as human memory can recall, its flooding regime is highly variable and spatio-temporally dynamic. As flooding in the LTCR varies significantly across time and space, this shapes inter-individual and inter-village variations in seasonal perceptions and experiences. While four seasons are broadly recognized by LTCR populations, the timing, duration and interpretation of seasonal shifts are often understood differently. In figure 2, a seasonal and activity calendar for each village (data averaged from individual interviews for villages in study one and from focus group interviews for villages in study two, with villages listed in order from northernmost to southernmost) shows the intervillage diversity in perception of seasonality and in activity patterns. At Impongui, a *terra firma* forest village that never floods, more than half of our respondents (*n* = 10) recognized only two or three seasons, omitting the small dry season most frequently. By contrast, Edzama residents, located around 20 km South and surrounded by hardwood swamp forest, most reliably listed all four seasons. More than half of the respondents there (*n* = 11), however, placed the small dry season during the months typically considered to be the long dry season (January to March). These collective intervillage data suggest that variability in flood timings (seasonality) becomes more pronounced further down the LAH.

**Figure 2.**
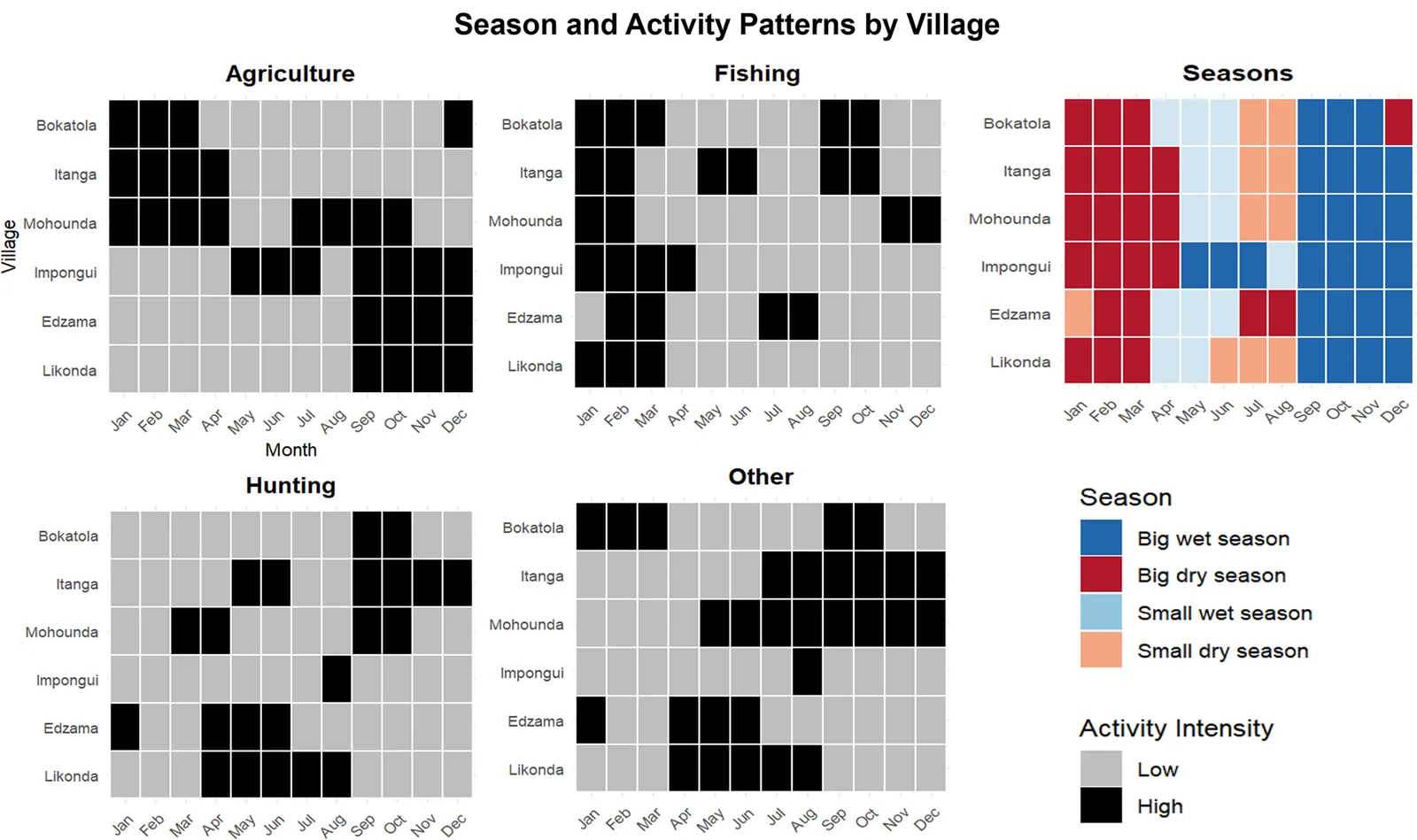
Seasons and activity budgets averaged per village. Plotted in colour is the relative timing of each season by month as recognized collectively by the residents of each study village. To the left of that plot, the intensity of livelihood activities is mapped, also by month and corresponding to each village's perception of seasonality. Activity category ‘Other’ mainly includes the collection/gathering of NTFP such as *Raphia* fronds, cola nuts and honey.

Village activity budgets represent which livelihood activities are predominantly practised during each season. We recorded livelihood activities as either ‘low’ or ‘high’ for the duration of each village's recognized seasons. While Reserve residents universally diversify their livelihoods relative to seasonality (next section), it is also important to observe the intervillage diversity present in figure 2. Likonda and Bokatola, while at opposite ends of our study area, seem to have comparably bifurcated activity cycles which probably reflects the fact that, of our study villages, these two experience the highest degree of seasonal flooding in and around their villages. Itanga, like Likonda, is located in an area of seasonally flooded forest, and these two villages have the most similar seasonal calendars between our northern and southern sites. However, unlike Likonda, Itanga is also proximal to the large strip of *terra firma* forest in the Reserve's centre. This provides its residents access to a more diverse array of ecological conditions and resource environments, which likely explains certain differences in activity budgets. Similarly, Mohounda and Impongui, both positioned on the large strip of *terra firma* forest, exhibit exceptionally divergent activity budgets, likely reflecting Impongui's proximity to the *terra firma*’s edge and thus their lesser reliance on seasonal hunting owing to year-round access to river and forest fishing areas. For all activities except fishing, there is a clear spatio-temporal contrast in activity timings across the North and South of the LTCR: agriculture, for example, is predominantly practised in the earlier months of the year in northern villages and towards the later months of the year in southern villages. Oppositely, hunting is predominantly practised in the earlier half of the year in more southern villages and later in the North.

We note that while figure 2 shows a correlation between physical geography and livelihood activity calendars, these patterns are outcomes of historically situated human–environment interactions, mediated by cultural knowledge, political memory and economic change—not products of ecological determinism [14]. While it is beyond the scope of this article to dive deeply into regional politics and cultural evolution, some of these relationships are evidenced by the ethnographic data presented below.

### (b) Local understandings of hydrological variability

There is further diversity in local understandings of how floods begin and then permeate the LTCR landscape. In the northern part of the Reserve, residents cite rainfall over forested areas as the primary source of flooding: ‘It takes maybe one week for the rains to reach here [Mohounda] from the Kabounga lands [region north of LTCR]. There are two canals there, one to the Moungouma Bailly [river] from Toukolaka, and another going to the Likouala-aux-herbes [river]. The water here comes from Cameroon via the Tonga river, which flows into the Likouala-aux-herbes near Matoko. The waters of the Oubangi flow directly into the Congo river. If it flooded there, it is going to flood here. The river never floods the village. It's mko [described as a type of sub-surface water that runs through underground channels] that floods it. It meets the water rising from the river. Mko flows from August onwards. You can see it at the village port, it's light-colored’ (Mohounda). In contrast to this quote, a respondent from Impongui, a village slightly further South, did not see rainfall as driving flooding: ‘the seasons are determined by [river] water level, not rainfall… There are different kinds of rain. One kind is when the rain begins at 5am and continues until late the next day.’ In Edzama, villagers understand the forest and water levels to rise at the same time, neither outpacing the other; the river rises to flood the outer band of riparian forest, while groundwater rises to saturate the inner swamp forests. Lastly, in Likonda, several people described an additional flooding regime where the river gradually rises and eventually floods its banks, funnelled into surrounding forest via terrestrial channels, ‘moloukou’ (not subterranean like the mko channels mentioned in Mohounda).

The forests surrounding Likonda, being seasonally flooded, boast *terra firma* ground. However, several respondents in this village insisted on their ground being distinct from the *terra firma* in the Reserve's central strip: ‘when floods leave the North, they fill the Cuvette where we are. At Likonda, we are at the bottom of the basin. Here we have *terra firma* but not the same* terra firma* as Impongui. Impongui is higher up in the basin […] Here, water only enters at our corners.’ Another Likonda respondent noted how, even right across the LAH from the village, the flooding regime and ecological communities are different: ‘on the other side of the river, there are different types of trees. It doesn't flood easily there; here the trees are aquatic […]; we don't have gorillas on this side of the river because their favorite foods [*Raphia* sp.] aren't here.’ The perception of a North-to-South flood gradient was common among respondents in our southern villages: ‘Floods start at Epena and trickle down. The North and South have opposite seasons’ (Likonda). Taken together with the data presented in figure 2 showing the intervillage variability in seasonal activity patterns, it is apparent that not only are local understandings and perceptions of flooding and hydrological processes across the LTCR tightly linked to livelihood cycles, but also that each village at LTCR is subject to specific flooding processes linked with micro-scale climatic and topological conditions, all corresponding to unique ecological assemblages.

The existence of distinct village- or area-specific lexicons reflects distinct village–environment relations and localized forest and water knowledge (supplementary material, table S2). For example, the word ‘*metele*’ (used in Bokatola) is a climatic anomaly that occurs at any time of year. It is a period during which the river waters are stagnant which has negative consequences for fishing. In contrast, ‘*miyela*’ (used in Itanga and Mohounda) occurs at the beginning of the short rainy season and is described as a series of back-and-forth river water movements, considered beneficial for fishing. In our southern villages, we did not encounter these terms and concepts, suggesting fine spatial variation in the hydrological processes of the region. These data show that LTCR residents display strong understandings of the regional-scale hydrologies driving local flood seasonality—‘Everything here is connected by rivers and floods, all the way to Cameroon’ (Edzama) – and simultaneously have fine-scale understandings of and adaptations to the diverse ways water moves across their landscape.

### (c) Remembering and recognizing ‘bad floods’

Significant flood events remembered included those in 1977, 2000, 2011 and 2020. In the South, respondents viewed ‘bad’ or extreme floods as occurring on roughly a 10-year cycle, although this statement was not shared as broadly in northern villages. Similarly, 1977 was not cited as one of the bad flood years in southern villages. As references to this flood year in the North were often accompanied by memories of State-sponsored aid (in the form of shipments, including goods such as mattresses, rice and oil), this North–South discrepancy may reflect political conditions. The southern district of Bouanela had yet to be formed and was much less accessible to administrators than Epena and the more northern reaches of the LAH. Moreover, many respondents perceived flooding in village areas as a relatively recent phenomenon. During the colonial formation of LTCR's villages, all were localized on ‘islands’ of dry land. Only since around the year 2000, respondents across villages assert, had flooding begun to breach village boundaries, reaching unprecedented spatial extents. As LTCR residents have only recently been exposed to extreme flooding within villages, this could explain why no LTCR natives have applied a common adaptation to seasonally flooded life—building housing on stilts—seen in communities inhabiting similar floodplain ecosystems across the tropics, including in peatland forests in the neighbouring Democratic Republic of Congo (for reference, see photo in CongoPeat [15, p. 21]). In further support of this assertion, it is common to see seasonal fishing camps located along the edges of the Mandoungouma River (a tributary of the LAH) built on stilted platforms above the water (figure 3), suggesting that LTCR inhabitants possess the capacity to construct on stilts but historically did not need to use it in village settings. Across study villages, residents mention a variety of techniques used to determine the severity of a flood event. The flooding of cassava fields—typically placed in the least flood-prone areas—signals a particularly bad flood. In Bokatola and Impongui, residents track the progression of floods by marking the river levels on the trunks of flooded riverside trees. In Edzama and Likonda, hunters relayed that they knew a bad flood had occurred when they found animals drowned in the forest. Across many villages, residents recalled the unprecedented need to use boats to traverse their village during the 2000 floods.

**Figure 3.**
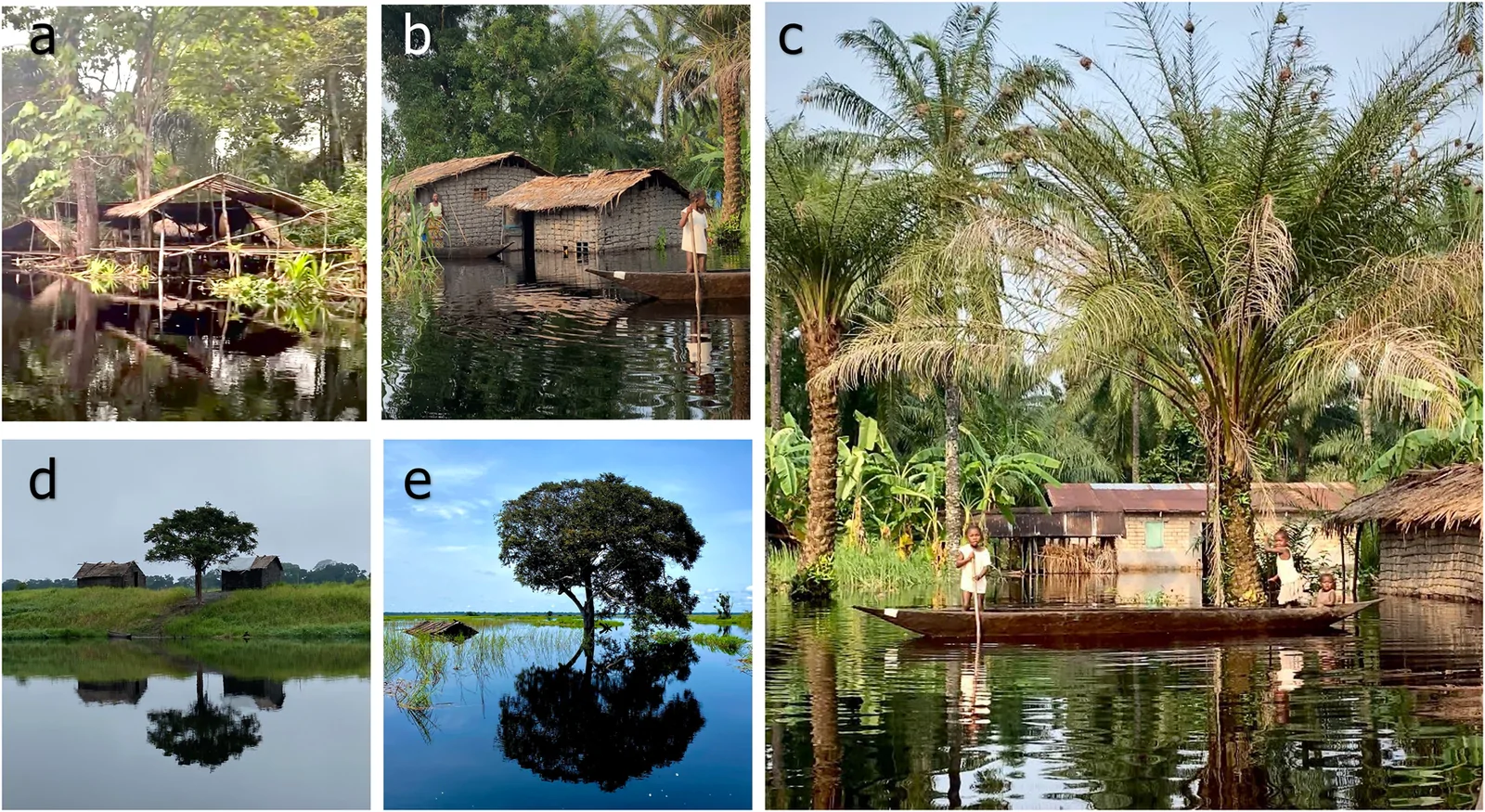
Examples of village and fishing camp flooding. (a) a seasonal fishing camp built on stilts along the edge of the Mandoungouma river (taken July, 2022). (b,c) Young children using a pirogue to travel between houses during flooding in the village Mabongo-Nkoto, northern LTCR (taken July 2023 during the unprecedentedly early big wet season). (d,e) Seasonal fishing camps along the LAH during low- and high-water periods. (Photo (d) taken March 2023. Photo (e) taken May 2023. Photos by K.M.)

### (d) Environmental and socio-political ramifications of flooding

Beyond ‘bad floods’, respondents underscored the deviation of present-day seasonal flooding patterns from historic cycles across all study villages (historic cycles corresponding to the broadly recognized small and big rainy seasons, with flood-waters never exceeding expected village limits such as cassava fields). Today, they say, floods are occurring more frequently, last longer and reach unprecedented levels. Our field period, 2023, featured one of the most exceptional flooding events in remembered history: ‘the water surprised us all’, said a Likonda resident of the floods that started in April 2023—four to five months ahead of schedule—‘we are in May; this should be the short rainy season but it is instead like the big rainy season!’ He went on to describe the far-reaching effects of seasonal and environmental change across the Reserve: ‘the river used to be very deep; boats could arrive to Epena even in the long dry season, now the river has lost its value […] it's now people who provoke the floods; there used to be respected burning periods. Now people burn however they want’ (Edzama).

This respondent emphasized a shift from natural floods to ones brought on by human practices, specifically burning land along the banks of the LAH. Traditionally, each village had a cultural leader, the *Notable* or *Ndami*, who regulated the timing and execution of important and often collective activities such as hunting, fishing and burning. Today, the Notable's influence is largely superseded by that of State-appointed village chiefs and weakens as more people reject traditional practices in favour of individualistic, income-generating activities. This shift, facilitated by the introduction of technologies such as headlamps that allow individuals to conduct unregulated practices more easily or covertly, was recognized by respondents in several villages who saw connections between increasingly unpredictable floods, corresponding droughts and uncontrolled burns of riparian and savanna areas (a practice that has a long local history and specific traditional regulatory processes [16]) which, in turn, leads to increased riverbank erosion and the filling in of the LAH. A shallower river and more extreme water recessions during low-water months mean never-before-seen impediments to river-based travel and associated trade on top of more fundamental challenges like lack of access to drinking water.

Today, as floods increasingly penetrate villages across the LTCR, flooding experiences shape distinct village identities and politics. In each of our three southern villages, residents insisted that the degree of flooding in their village was unique compared to surrounding ones, lending them superiority or advantage, particularly regarding access to dry land. Likonda residents compared themselves to residents of Edzama, Bondoki and Epena: ‘Edzama floods! Where we live, that's a ruisseau [see table 1]. It's different’; ‘Epena floods but here in Likonda, it's only in the corners. At Epena, you go live with your neighbors during floods [*…*] At Bondoki, their forest has water everywhere. Here is the dry forest’; ‘We don't flood here. In the forest it floods but our fields don't. Other villages come to use our fields.’ Edzama residents noted how, despite their greater susceptibility to flooding, the predictability of floods and the importance of ancestral connections to land made life there tenable: ‘How do we survive? We are from here. It's our ancestral home. Edzama doesn't flood randomly like it does at Kinami, for example’; ‘Our ancestors left us here. We cannot leave, we must respect tradition. You can always find dry land to live on.’ Impongui residents compared themselves to Epena: ‘the difference between Epena and Impongui is we can cultivate our fields during the flooded period. At Impongui, life is better and simpler because there are no floods’; ‘At Botongo and Mosingi, people were displaced by floods in 2000 but we were able to stay.’ In each village across the LTCR, cultural and spiritual ties to ancestral forest and river territories keep people rooted to seasonally flooded landscapes, while intervillage mobility provides a means to withstand particularly severe flood events.

### (e) The river–peatland network and its value to local livelihoods and cultures

Since seasonal flooding in flood-pulse river systems like LTCRs drives the fine-scale spatio-temporal distribution of ecological diversity and productivity, the dynamics of flooding across the LTCR landscape exert significant influence on what resources are available to Reserve residents, when and where. LTCR residents exhibit Reserve-wide adaptations to their uniquely water-dominated environment, including diverse, seasonal and flood-dependent livelihoods and cultural practices.

#### (i) Ancestral lands

LTCR inhabitants have highly mobile yet territorial relationships to places. From an early age, children are trained in essential water-based survival skills, including swimming and the operation of dugout canoes, which are integral to transportation and subsistence activities (figure 3). Canoes are typically custom-made, with paddles designed to individual specifications. Across the LTCR, settlement distribution is primarily governed by ancestral land tenure. Asentiment similar to that expressed above by the Edzama resident about enduring floods due to ancestral ties to place, is widely expressed in all study villages, including those subjected to more intense floodings, such as northern Bokatola. Itanga residents, for example, collectively decided to relocate in 1962—spurred by factors including the water stagnation within village grounds, challenge of needing to use pirogues for basic travel and deterioration of most earth-based homes brought about by prolonged flooding. They did so, however, only within the limits of their ancestral territories (see [16, p. 68], for a visualization of village ‘*terroirs*’). In several villages, respondents described the historical practices of using termite mound deposits (called ‘*bangoumou*’ in central areas; see supplementary material, table S2) to elevate housing foundations and maintain dry zones for hearth placement. This method remains in use, albeit progressively substituted by contemporary construction using materials such as bricks, used to elevate living spaces, furniture or beds. In Bokatola, respondents reported that a minimum height of four bricks was now considered essential to mitigating flood impacts.

#### (ii) Agriculture

All observed households maintain diversified livelihood portfolios that are closely synchronized with hydrological variability, integrating agriculture, fishing, hunting and NTFP collection. Agricultural practices are both spatially and temporally structured around flood dynamics. Respondents involved in agriculture (mostly women) described a prioritization approach to cassava harvesting, wherein fields most vulnerable to flooding are harvested first, while crops in drier zones are left to mature longer. In areas subject to frequent and severe flooding, all cultivated fields are dedicated to short-cycle crop varieties to mitigate potential losses. A small number of respondents in northern villages also mentioned the historical use of raised agricultural fields as a flood adaptation strategy. While informants often cited flooding as a limiting factor for cassava cultivation owing to crop loss and restricted field availability, it is simultaneously recognized as an essential agroecological process for nutrient cycling and soil fertility restoration.

#### (iii) Fishing

Flood variation and corresponding fish movement and reproductive ecologies also dictate subsistence and commercial fishing patterns. Fishing techniques are highly stratified by flood phase and vary across the LTCR with techniques adapted to fluctuating water levels and fish migration patterns. The big dry season constitutes the principal fishing period, as fishers capitalize on flood recession to maximize catches in both the river and savanna/forest pools. During this period, men and women temporarily leave the village to engage in intensive fishing operations at family-owned fishing camps, located either along the main river channels or within the forests. In Likonda, several respondents mentioned the near abandonment of the village during low-water periods: ‘sometimes only five or six people remain in the village. Animals then aren't scared away by the noise of the village. One time, a village dog was eaten by a python that came right into the village.’ The big dry season represents a critical economic period, characterized by heightened fishing activity and expanded trade networks, increasingly integrated into regional and national commercial circuits. A poor big dry season, characterized by low fish catches owing to inadequate water recession, can severely impact household finances, as many rely on fishing income to buy food staples such as oil and salt, cover education costs, access healthcare and buy essential household goods like soap. A diversity of fishing techniques, employed in accordance with seasonal shifts, were mentioned by respondents, with cross-village variability. During the big dry season, when water levels are low, fishers employ *kokopa*, a manual removal of water from ponds to concentrate fish, a practice historically performed with the *ekoumba*, a tub woven out of *Raphia* and operated by four individuals. Women commonly used *eyika Raphia* baskets (Itanga) in small ponds and riverbanks, alongside *mileke* and *molo* fishing traps. Other communal techniques include the *okala* trap, used to capture fish, caimans and snakes in rivers and large ponds, as well as large *motresse* nets. *Mbilo* fishing involves clearing vegetation to channel fish into designated catchment areas. During the big rainy season, when water levels are high, fishers shift to *ndobo* hook and line fishing, a technique for individual subsistence. Nets remain in use year-round but are adjusted in mesh size and placement based on water levels and fish migration routes.

#### (iv) Hunting and gathering

Seasonal floods expand people's access to remote and otherwise inaccessible areas within their territories. Thus, in high-water seasons, hunting and gathering of staple and commercial forest resources increase. Hunting activities across Reserve villages traditionally peak during the high-water season, when flooded landscapes concentrate wildlife populations, making tracking and capture more efficient. The dry season, however, can also facilitate hunting in areas adjacent to water sources. Our interlocutors described historical hunting with spears and nets (*bokila*), often requiring group or village-wide coordination to capture game. Similar to the gradual deregulation and devolution of village burning regimes, hunting has become a more individualistic and unregulated behaviour, particularly following the influx of firearms into the region following the Republic of the Congo Civil War (1997–1999) and other armed conflicts in neighbouring Central African Republic and Democratic Republic of Congo [17]. Water levels also influence logistics and material transport, creating temporary waterways that facilitatemovement across the landscape. Seasonal shortcut routes, often named after significant locations, facilitate trade, family visits and participation in cultural rites. Seasonal floods, most importantly, are widely seen as sustaining  an ecological ‘equilibrium’, allowing animal populations, fish especially, time and space to reproduce. Though fish are harder to find during the flooded season when they spread out across the watery landscape, fish from the forest are considered more nutrient dense than those caught in the LAH. Moreover, this period also corresponds to a high production period for other staple food resources such as safu (*Dacryodes edulis*, a fruit tree species native to the Congo Basin) and edible caterpillars. Transporting of heavy construction materials (specific species of hardwood trees) is made easier by water-based travel through flooded forest areas, and the duration of travel times between villages (by boat along the LAH) is greatly reduced during the flooded season, facilitating intervillage social and material exchange.

#### (v) Trade and new markets

In northern villages, respondents emphasized the historical role of food preservation technologies to mitigate periods of food scarcity during peak flood periods. Traditional fish preservation methods include smoking or salting and sun-drying. Cassava, meanwhile, is fire-dried after peeling off the outer skin, placed in woven palm baskets (*mokota* in Itanga) and submerged in the river for gradual consumption. In central villages, large storage jars (*ediba*, referred to as ‘freezers’) were used for both fish and cassava. Nowadays, traditional food preservation practices are in decline, as any surplus is sold for cash rather than stored: ‘conservation was an old practice; these days, we sell everything’ (Bokatola). However, with increasing market integration, new materials and methods for food acquisition and preservation are emerging. In several of our study villages, fishing with soap was said to be a practice increasingly taken up by village youth and non-local fishermen coming from across the border with the Democratic Republic of Congo, allowing them to catch more fish at one time but contaminating the surrounding water in the process. In southern villages, we also observed fish sellers in multiple villages spraying their mallets of smoked fish—destined for trade as far as Brazzaville and into surrounding Central African countries—with a high-potency herb/pesticide called ‘Sniper’ that, in other nearby countries, has become popular as a means of suicide. More LTCR residents are prioritizing short-term commercial gain as flooding patterns and resource availability become less predictable, synergizing with deteriorations in traditional resource governance mechanisms and socio-economic conditions.

Overall, respondents expressed conflicted sentiments over their lives and livelihoods in LTCR's seasonally flooded swamp environment. Water's predominance across the LTCR socioecological system presents both benefits and challenges. On the one hand, residents feel well-adapted to a rich ecosystem, having inherited and refined a wide range of livelihood strategies and having access in each village territory to diverse microhabitats and associated natural resources and processes. On the other hand, many voiced concerns about the increasing unpredictability of floods: ‘the water forces us to work at a certain pace, and there are the disruptions of the seasons’ (Itanga). Beyond hydrological uncertainty, respondents highlighted broader socioecological shifts, including the intensification of natural resource harvesting, increased fire frequency and the erosion of customary practices, including collective resource governance, reshaping traditional food security strategies. In Bokatola, for example, a pond once considered spiritually significant is now actively exploited: ‘it used to be a site of power, but now we neglect those traditions.’ A respondent in Impongui noted how the colonial introduction of cacao cultivation—an economy that LTCR managers are now attempting to revitalize as an alternative livelihood project in several villages—drove the loss of traditional forest fishing practices: ‘we used to fish in forest pools during the big dry season but this practice doesn't exist now because of the introduction of cacao. Now during that season, people need to arrange their cacao fields, then harvest the fruits in the big rainy season.’

## 4. Discussion

This study represents one of the first ethnographic investigations—incorporating qualitative, semi-structured data and prolonged participant observation—in the LTCR. Up to now, the small body of research pertaining to the LTCR includes (mostly unpublished) colonial-era reports documenting aspects of Bomitaba culture along the LAH river (e.g. [18]), landscape-scale institutional reports (e.g. [19]) and sporadic academic works which have largely focused on the Reserve's more accessible northern and central sectors and engaged either ecological or quantitative survey methods [10,11,16,20–22]. Our ethnographic work captures a more complete spectrum of socioecological dimensions within the LTCR from North to South and is distinctive in its method and scope within the Congo Basin peatland literature (cf. [23,24]). Moreover, encompassing six distinct villages across the 4400 km² Reserve, our work is unusual in its geographic scope among the broader human–wetland scholarship from around the world [4,25–33].

Our findings reveal both overarching patterns and fine-scale variations in how communities within the LTCR interpret and experience flooding seasonality. Annual flooding exhibits spatial and temporal heterogeneity, generally progressing along the LAH river from North to South but penetrating each village–forest system in unique ways. During the colonial period, each village was established around small areas of unflooded ground amidst the larger seasonally flooded peat swamp ecosystem. Since around the year 2000, however, LTCR residents have experienced increased floods that inundate their villages partially or completely. At LTCR, livelihoods are sustained through flexible, multimodal subsistence, with households reallocating labour in response to the annual flood pulse. While the dry season is preferred for food security, fish being more easily available, the flooded season is valued for house construction and forest-based fishing. Floods characterized as ‘bad’ result in the inundation of typically dry areas such as cassava fields, villages and homes. Mobility serves as a key adaptation, though ancestral territorial ties limit permanent relocation. Other behavioural adaptations to floods exist heterogeneously across villages, including using termite mounds to elevate housing or agricultural beds, paralleling practices in other floodplain communities neighbouring the Bomitaba [34,35]. Recently, flooding patterns have shifted with earlier flood onset and prolonged droughts. Despite the challenges, seasonal floods are widely recognized as ecologically beneficial, supporting fish populations and nutrient distribution. Historically and until present days, intervillage variations in flood knowledge and experiences have been largely influenced by fine-scale hydrological dynamics: while some villages remain unaffected by floods, others experience annual inundation or extreme flooding, particularly in the northern sector. This results in differential flood vulnerability across villages, as well as divergences in flood memory.

Across our identified themes, accelerating socioecological changes across the Reserve is evident, shaped by both climatic and socio-economic shifts. A decline in traditional governance structures has led to the erosion of communal resource management practices, including burning, hunting and fishing (e.g. [16]). Market integration increasingly shapes local livelihoods, pushing local communities to seek increased income sources and leading to, for example, increased forest clearing for commercial manioc. Unsustainable fishing practices (e.g. fishing with soap or spraying pesticides on smoked fish exports) yield short-term economic benefits but contribute to long-term environmental degradation. Those socioecological changes are further amplified by disruptions in the LTCR seasonal flood pulse. Our observations suggest that long-standing adaptive frameworks within LTCR communities are under increasing strain owing to climatic shifts, economic transitions and the erosion of knowledge systems. While demographic variables, such as age, gender and occupation influence individual observations related to flooding and environmental change, a detailed examination of these factors falls beyond the scope of this study. However, though the social and ecological stressors faced by local communities across the LAH are relatively universal, experiences of and responses to them are uneven and situated within diverse socio-political and environmental histories, highlighting the importance of approaching each village-forest territory at LTCR as a unique ‘hydrocultural patch’.

### (a) The ‘hydrocultural patch’: policy and governance implications

Our work, like that of other social scientists working in the Congo Basin peatlands [36], emphasizes the socioecological compexity of the Congo Basin peatlands: hyper-diversity and high levels of temporal dynamism in biocultural conditions across relatively small areas. However, as research on this region rapidly advances, emphasis is being placed on methods that produce landscape-scale or basin-wide results that can be easily translated into sweeping policy recommendations. These approaches create a mismatch with the results of social scientists. Thus, when debating at what scale to study and/or govern these river-floodplain forests and their inhabitants, there is a need for a framework that bridges the methodological and theoretical divides between social and natural scientists. We argue that rivers and flooding present such a bridge. River ecologists recognize that river-floodplain networks can be broken up into distinct (but continuous) ‘hydro-geomorphic patches’, each with their own ‘size, vertical and lateral shape, internal structure/hydrological complexity [and other components of] biocomplexity’ [37, p. 126]. This complexity—driven by the flood pulse—in turn determines the system's productivity and ecological diversity, favouring human resource-use patterns or livelihoods that are equally diverse and seasonally flexible [13, p. 124]. However, this relationship between riverine biocomplexity and human livelihoods is a complex and reciprocal one. Social scientists studying the human–fish interface across two riverine village communities in Central Kalimantan, Indonesia, have furthered a 'biocultural' research framework to study and conserve the broader peatland and wetland landscapes [4]. They define biocultural diversity as ‘the dynamic, place-based multiplicity of human and nonhuman beings, their livelihoods and their constituting relations’ [4, p. 349], showing how a focus on such relationships, rather than nature or culture alone, could herald more effective and just conservation outcomes. Our findings support combining these transdisciplinary understandings of riverine or wetland systems, suggesting the extension of the hydro-geomorphic patch concept to include contingent biocultural variation—a division of the LAH, for example, into discrete but relational ‘hydroculturalpatches’. The idea of the ‘hydrosocial’ has been used by several critical scholars working in water-dominated or hydrologically important environments, particularly as a way to emphasize the role of politics and power in shaping water governance, centering themes of social and multispecies justice [38–40]. Our term combines these ideas to encompass how diversity is arranged by both natural, cultural, and political forces in water-dominated systems. While the Congo Basin peatlands may be a single hydrological unit, approaching their governance as such erases the water-driven biocultural diversity that exists at scales so fine as to obscure the straightforward characterization of even a single river catchment or protected area such as the LTCR.

Recognized as ‘natural climate solutions’, wetland ecosystems, particularly peatlands, are increasingly at the centre of scientific concern amid the global climate crisis [25,41]. However, their unique hydrological dynamics make them highly vulnerable to Anthropocene-driven changes. This growing fragility has intensified the urgency to understand and value the world's remaining wetlands, particularly those of the Congo Basin. Pursuing a singular, climate-centric research agenda, however, risks oversimplifying or neglecting the fine-scale human–environment interactions that both shape and are sustained by wetland ecosystems. This reductionist approach to research and the resulting top-down policies that it informs have been shown in other wetland systems to have negative impacts on socioecological resilience, such as reduced adaptability and greater uncertainty during extreme flooding events [42]. Addressing the mounting challenges facing these peatlands and their human and non-human communities will necessitate a hybrid conservation approach that integrates local ecological knowledge with scientifically informed environmental governance [43,44]. Globally, conservation scientists are calling for research and policy development that is place-based, co-produced and adaptive [45–47], particularly in water-dominated systems like wetlands where system components are uniquely entangled and threatened [40,48,49]. Our work answers these calls and contributes to a growing body of literature on human–peatland interactions in the Congo Basin [36]. It furthermore builds on existing work in conservation science to integrate social and natural science-based ways of knowing and valuing critical ecological landscapes [50]. Ethnographic research on topics of scientific concern, thus, should be systematically integrated into research priorities, funding allocation and policy development. Recognizing that the socio-cultural value of the Congo Basin's peatlands is tightly linked to and as spatio-temporally diverse as its fine-scale hydro-ecologies, we suggest that the ‘hydroculturalpatch’ be considered an essential unit (village-level) and method (transdisciplinary, incorporating ethnography) of analysis for researchers seeking to produce data of relevance to policy and governance development. As the social and ecological challenges facing the Congo Basin peatlands are so clearly intertwined, taking such a fine-scale, human-oriented approach will allow researchers and policymakers to recognize where the most important synergies are between the diverse human and non-human communities they seek to support through conservation action.

## Supplementary Material



## Data accessibility

Our ethnographic data come from interviews and focus groups conducted under the condition of anonymity. Therefore, we cannot share the transcript data publicly.

## Theme

One contribution of 17 to a discussion meeting issue ‘African tropical peatlands: function, value and vulnerability’.
