# Supplementary material for: Fine-scale human–flood relationships in the Congo Basin: ethnographic perspectives from the Lac Télé Community Reserve
Source: Philos Trans R Soc Lond B Biol Sci. 2026 Aug 20;381(1956):20240491. doi: 10.1098/rstb.2024.0491 (PMC13732396; doi:10.1098/rstb.2024.0491)
Supplement: Supplementary file 1 [file rstb.2024.0491.zip › rstb.2024.0491/rstb.2024.0491/rstb20240491_si_001.pdf]

Fine-scale human-flood relationships in the Congo Basin: ethnographic perspectives from the Lac Télé Community Reserve

Katherine Meier, Milena Beekmann, Tiriel Lokoka Ngoma, Célie-Léoda Moungouya-Moukassa, Bola Madzoke

Table 1. Study village details. Socio-ecological and research contexts ("Population size" and "Dominant ethnic group" data derived from Bondeko et al., 2016).

Village	Reserve location	Political district	Research team	Flooding regime (during high-water season)	Adjacent forest type	Population size	Dominant ethnic group
Bokatola	North	Epena	MB/CM	Fully flooded	Hardwood swamp forest	222	Nzobo (98%)
Mohounda	Center North	Epena	MB/CM	No flooding	Dry forest	252	Babolé (100%)
Itanga	Center	Epena	MB/CM	Partially flooded	Seasonally flooded forest	343	Baitanga (90%)
Impongui	Center South	Epena	KM/TLN	No flooding	Dry forest	155	Babolé (94%)
Edzama	South	Bouanela	KM/TLN	Partially flooded	Hardwood swamp	425	Babolé (92%)
Likonda	South	Bouanela	KM/TLN	Partially flooded	Seasonally flooded forest	373	Babolé (97%)

Table 2. Glossary of relevant Bomitaba terminology relevant to local hydro-climactic variables. Derived from semi-structured interviews, this glossary shows the diversity in the types of water, land, and other environmental variables recognized by local communities across the Reserve, but also the cultural (linguistic) diversity evidenced by the multiple village or locality-specific iterations of a single word. In word descriptions for "Ewassa" or "Moloukou," for example, it is also clear that local understandings of these types of land/water are linked to livelihood activities such as fishing and cutting manioc fields.

**Glossary of Bomitaba terms**			
**Bomitaba word**	**French equivalent**	**Meaning / description from interviews**	**Villages/localities where term was heard**
**Seasons**			
Muanga/Tamba Nfâ	Grande saison sèche	Big dry season: typically falling between January and April	Muanga (all except...), Tamba Nfâ (Bokatola)
Mpela/Soba	Grande saison des pluies	Big wet season: typically falling between September and January	Mpela (all except...), Soba (Bokatola)
Ndjobolo/Essebo	Petite saison des pluies	Small wet season: typically falling between April and June	Ndjobolo (all except...), Essebo (Bokatola)

*Author for correspondence (katherine.meier@yale.edu).
†Present address: Department of Anthropology, Yale University, 10 Sachem St., New Haven, CT 06511, USA

Fine-scale human-flood relationships in the Congo Basin: ethnographic perspectives from the Lac Télé Community Reserve

Katherine Meier, Milena Beekmann, Tiriel Lokoka Ngoma, Célie-Léoda Moungouya-Moukassa, Bola Madzoke

Essebo/Ekongo	Petite saison sèche	Small dry season: typically falling between June and September	Essebo (all except...), Ekongo (Bokatola)
**Types of land and water**			
Ewassa	Forêt saisonnièrement inondée	*"Example at the river when you get to the plain - forest where water enters easily;" "where water enters"; "an open forest that floods (large compared to Moloukou)"; "a terre ferme (no mud) but water enters easily, like near the port"; "a seasonally flooded area"*	All
Mokolo		"*To the North*"; "*an area that doesn't flood*"	Meaning "an area that doesn't flood" in the South, otherwise meaning "to the North"
Eboungo	Vasière	"*A muddy area*"; "*a swamp*"	All
Moloukou/Mobanda/Mololou	Ruisseau	*"Where you might go fish"; "where you enter forest and go all the way to where there are fish"; "places you go to put your nets"; "open forest, often between two areas of terre ferme"; "a seasonal passage for water"; "the route we use to access fields"; "in between two forests"; "the passage for water in the forest"*	Moloukou (Likonda), Mobanda (Mohounda/Boha), Mololou (South)
Bonguta	Savane		All
Moungondo/Mokondo	Terre ferme	*"Where it doesn't flood"; "where we cut fields"*	Mougondo (North), Mokondo (South)
Metele		Stagnant water, a climatic anomaly during any season	Bokatola
Miyela		Back-and-forth river water movement recognized to occur at the beginning of the short rainy season; considered beneficial for fishing	Itanga/Mohounda
Dissawou	Savane inondable	Flooded savanna	Mohounda
Mko		Subterranean water that flows out of the forest through underground channels into the main river system at the beginning of the high-water season (August- December); light-colored water that mixes with river water during flooding	Mohounda

*Author for correspondence (katherine.meier@yale.edu).
†Present address: Department of Anthropology, Yale University, 10 Sachem St., New Haven, CT 06511, USA
