# Supplementary material for: Fine-scale human–flood relationships in the Congo Basin: ethnographic perspectives from the Lac Télé Community Reserve
Source: Philos Trans R Soc Lond B Biol Sci. 2026 Aug 20;381(1956):20240491. doi: 10.1098/rstb.2024.0491 (PMC13732396; doi:10.1098/rstb.2024.0491)
Supplement: Supplementary file 1 [file rstb.2024.0491.zip › rstb.2024.0491/rstb.2024.0491/rstb20240491_si_002.pdf]

# Relations entre les populations et les inondations à l'échelle locale dans le bassin du Congo : perspectives ethnographiques de la Réserve Communautaire du Lac Télé

Katherine Meier, Milena Beekmann, Tiriel Lokoka Ngoma, Célie-Léoda Moungouya-Moukassa and Bola Madzoke

https://doi.org/10.1098/rstb.2024.0491

*Mots-clés : Écosystèmes inondés saisonnièrement, Inondations extrêmes, Communautés riveraines, Savoirs écologiques locaux, Adaptation au changement climatique*

La rivière Likouala-aux-Herbes et les forêts marécageuses environnantes du nord de la République du Congo sont décrits dans la littérature scientifique comme un système forestier humide caractérisé par un régime de crues bimodales accompagné d'inondations saisonnières sur des vastes étendues. De récents modèles climatiques et hydrologiques du bassin du Congo et des tourbières de la Cuvette Centrale présentent en outre la région de la Likouala comme une tourbière ombrotrophe, ou alimentée principalement par les précipitations. Les populations humaines sont cependant confrontées aux inondations et s'y adaptent à une échelle plus locale. Cette étude présente des données ethnographiques sur les inondations et la saisonnalité recueillies dans six villages de la Réserve communautaire du Lac Télé (LTCR), une aire protégée située dans le biome des forêts marécageuses de la Likouala et seule réserve communautaire du pays. Nos résultats démontrent comment les inondations et leur variabilité spatio-temporelle engendre des cultures, des cycles de subsistance, des formes d'adaptation et des vulnérabilités propres à chaque village - un gradient interconnecté de relations bioculturelles. Dans tous les villages, la dynamique des inondations a considérablement évolué au cours des dernières décennies. Le changement climatique, les transitions économiques et l'érosion des systèmes de connaissances locales compromettent les mécanismes d'adaptation établis de longue date au sein des communautés LTCR, ainsi qu'entre elles. Nous soulignons l'importance des données ethnographiques pour comprendre les interactions sociétés humaines-environnement et suggérons que l'analyse bioculturelle à cette échelle – ce que nous appelons la « parcelle hydro-sociale » – est essentielle pour une gouvernance paysagère efficace et l'élaboration de politiques dans le contexte de l'accélération du changement climatique.
